# The *TMEM189* gene encodes plasmanylethanolamine desaturase which introduces the characteristic vinyl ether double bond into plasmalogens

**DOI:** 10.1073/pnas.1917461117

**Published:** 2020-03-24

**Authors:** Ernst R. Werner, Markus A. Keller, Sabrina Sailer, Katharina Lackner, Jakob Koch, Martin Hermann, Stefan Coassin, Georg Golderer, Gabriele Werner-Felmayer, Raphael A. Zoeller, Nicolas Hulo, Johannes Berger, Katrin Watschinger

**Affiliations:** ^a^Institute of Biological Chemistry, Biocenter, Medical University of Innsbruck, A-6020 Innsbruck, Austria;; ^b^Institute of Human Genetics, Medical University of Innsbruck, A-6020 Innsbruck, Austria;; ^c^University Clinic for Anesthesiology and General Intensive Care Medicine, Medical University of Innsbruck, A-6020 Innsbruck, Austria;; ^d^Institute of Genetic Epidemiology, Department of Genetics and Pharmacology, Medical University of Innsbruck, A-6020 Innsbruck, Austria;; ^e^Department of Physiology and Biophysics, Boston University School of Medicine, Boston, MA 02118;; ^f^Institute of Genetics and Genomics, University of Geneva, 1211 Geneva 4, Switzerland;; ^g^Department of Pathobiology of the Nervous System, Medical University of Vienna, 1090 Vienna, Austria

**Keywords:** plasmalogen, transmembrane protein 189, plasmanylethanolamine desaturase, ether lipid

## Abstract

A significant fraction of the glycerophospholipids in the human body is composed of plasmalogens, particularly in the brain, cardiac, and immune cell membranes. A decline in these lipids has been observed in such diseases as Alzheimer’s and chronic obstructive pulmonary disease. Plasmalogens contain a characteristic 1-*O*-alk-1′-enyl ether (vinyl ether) double bond that confers special biophysical, biochemical, and chemical properties to these lipids. However, the genetics of their biosynthesis is not fully understood, since no gene has been identified that encodes plasmanylethanolamine desaturase (E.C. 1.14.99.19), the enzyme introducing the crucial alk-1′-enyl ether double bond. The present work identifies this gene as transmembrane protein 189 (*TMEM189*). Inactivation of the *TMEM189* gene in human HAP1 cells led to a total loss of plasmanylethanolamine desaturase activity, strongly decreased plasmalogen levels, and accumulation of plasmanylethanolamine substrates and resulted in an inability of these cells to form labeled plasmalogens from labeled alkylglycerols. Transient expression of TMEM189 protein, but not of other selected desaturases, recovered this deficit. TMEM189 proteins contain a conserved protein motif (pfam10520) with eight conserved histidines that is shared by an alternative type of plant desaturase but not by other mammalian proteins. Each of these histidines is essential for plasmanylethanolamine desaturase activity. Mice homozygous for an inactivated *Tmem189* gene lacked plasmanylethanolamine desaturase activity and had dramatically lowered plasmalogen levels in their tissues. These results assign the *TMEM189* gene to plasmanylethanolamine desaturase and suggest that the previously characterized phenotype of *Tmem189*-deficient mice may be caused by a lack of plasmalogens.

## Significance

Although sequencing of the human genome was completed years ago, we still do not know about the physiological significance of thousands of predicted proteins, particularly of predicted membrane proteins. On the other hand, for approximately 100 human enzymes, no coding gene is known even though their enzymatic reaction has been well characterized. In this work, we assign one of those predicted membrane proteins (transmembrane protein 189; TMEM189) to one of the enzymatic reactions with an uncharacterized gene (plasmanylethanolamine desaturase). This enzyme catalyzes the final step in the biosynthesis of plasmalogens, an abundant class of glycerophospholipids that is depleted in such diseases as Alzheimer’s. Our findings enable interpretation of the previously characterized impaired growth phenotype of *Tmem189*-deficient mice.

Plasmalogens are a special type of glycerol-based phospholipids that are abundant in human and animal bodies. Yeast, aerobic bacteria, plants, and most nonanimal organisms do not contain this special glycerophospholipid class. Interestingly, some anaerobic bacteria also form plasmalogens using enzymatic reactions different from those in animals (1). In humans, plasmalogens constitute roughly one-fifth of all phospholipids, with particularly high concentrations in brain and in immune cell membranes (2). A decline in plasmalogens has been associated with Alzheimer’s disease (3–6) and autism spectrum disorders (7). Plasmalogens are important constituents of surfactants in the lung and have been shown to effectively reduce the surface tension of surfactant-like phospholipid mixtures (8). It has been suggested that decreased plasmalogen levels in smokers could be involved in the development of smoking-related diseases (9).

**Fig. 1.**
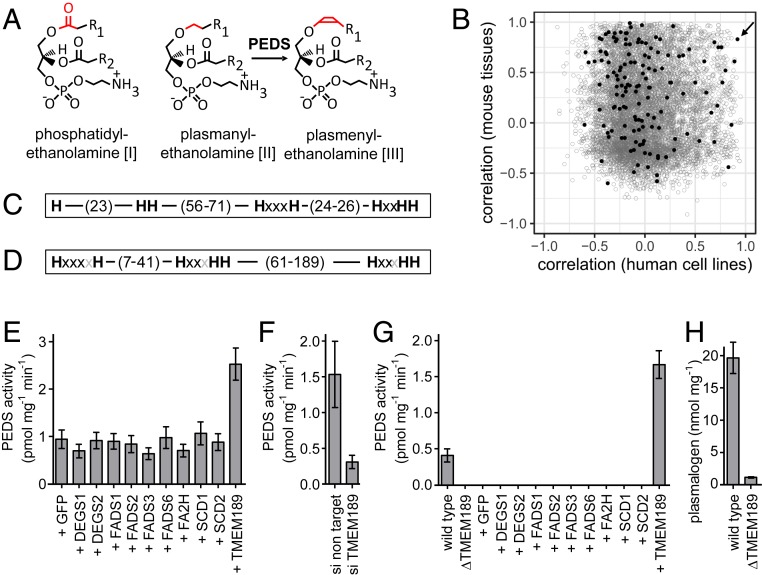
PEDS gene identification. (*A*) Formulas of phosphatidylethanolamine [I], plasmanylethanolamine [II], and plasmenylethanolamine [III], which differ in their bonding type at *sn*1 (red). The PEDS reaction that generates plasmalogens by introduction of the 1-*O*-alk-1′-enyl double bond is indicated by an arrow from [II] to [III]. R_1_ and R_2_ denote the typical hydrocarbon linear side chains of mammalian lipids comprising (in addition to the two side chain carbons shown in the formula) typically 14 or 16 carbon atoms and zero or one double bond (R_1_) and approximately 14 to 22 carbon atoms and one to six double bonds (R_2_). (*B*) Pearson correlation coefficients of PEDS enzymatic activities (22) with normalized mRNA sequence counts for 7,382 genes commonly expressed in cells or tissues with PEDS activity downloaded from NCBI GEO datasets for seven mouse tissues (*y*-axis) and 11 human cell lines (*x*-axis). Black solid circles, genes with gene symbols with Tm as the first two letters, which are mostly transmembrane proteins with unknown function; gray open circles, other genes. The arrow indicates *TMEM189*. (*C*) Schematic representation of the conserved histidines deduced from a sequence alignment of the 10 most diverse members of the pfam10520 protein family as displayed in the NCBI conserved domain database (CCD) (43). (*D*) Schematic representation of the classical eight-histidine motif of stearoyl-CoA desaturase and related desaturases (23). (*E*) PEDS activities of human HEK293T cells at 48 h posttransfection with selected expression plasmids for desaturase and hydroxylase proteins compared with a TMEM189 protein expression plasmid. GFP (green fluorescent protein) served as a transfection efficiency control. Data are mean ± SEM of four independent experiments. (*F*) PEDS activities in human A431 cells treated for 72 h with siRNA pools. Data are mean ± SEM of five independent experiments. (*G*) PEDS activities in WT HAP1 cells compared with *TMEM189*-deficient HAP1 cells (∆TMEM189) at 48 h posttransfection with expression plasmids for GFP, for selected desaturase and hydroxylase proteins, and for TMEM189 protein. Data are mean ± SEM for three independent experiments. (*H*) Plasmalogen content of WT HAP1 cells compared with *TMEM189*-deficient HAP1 cells. Data are mean ± SEM for three independent experiments.

Rare inherited disorders have been characterized that impair the initial peroxisomal steps of plasmalogen biosynthesis, resulting in rhizomelic chondrodysplasia punctata types 1 to 5 (10) with severe clinical outcomes, including impaired neural development, bone deformation, and premature death. The initial peroxisomal steps of human plasmalogen biosynthesis have been well characterized to require the genes *GNPAT* (11, 12), *AGPS* (13), and *DHRS7B* (14). Further downstream steps occurring in the endoplasmic reticulum are less well understood, however. Enzymes modifying the *sn*2 and *sn*3 positions during the biosynthesis of ether lipids in the endoplasmic reticulum might be shared to an unknown extent between ether and ester-linked glycerophospholipids. Recently, *SELENOI*, which encodes ethanolamine phosphotransferase 1, has been found to play a crucial role in plasmalogen biosynthesis (15); however, the gene coding for the enzyme generating the first plasmalogen in the pathway, plasmanylethanolamine desaturase (PEDS, E.C. 1.14.99.19), has not yet been identified. This enzymatic reaction is specific for plasmalogen biosynthesis. It introduces the alk-1′-enyl ether double bond (vinyl ether bond) into plasmanylethanolamines, yielding plasmenylethanolamines (Fig. 1*A*), the first plasmalogens formed in the biosynthetic pathway (2). In contrast, plasmenylcholines (plasmalogens of the phosphocholine head group class) are not substrates of the enzyme and thus must be synthesized from plasmenylethanolamines (2).

Importantly, the alk-1′-enyl ether double bond confers crucial biophysical, biochemical, and chemical properties to plasmalogens (6, 16). Plasmenylethanolamine has been shown to have a dramatic impact on the structure of phospholipid bilayers (17) and to facilitate rapid membrane fusion (18). Both properties are not shared by the corresponding ester-linked phosphatidylethanolamines (Fig. 1*A* shows chemical structures). Due to the alk-1′-enyl ether double bond, plasmalogens become sensitive to chemical cleavage by low concentrations of ozone (19) or by hydrochloric acid (6), resulting in formation of an aldehyde. The liberation of an aldehyde on acid treatment resulted in the detection of these compounds, giving rise to their designation as plasmalogens (aldehyde-releasing compounds found in cell plasma) (20).

Here we present evidence that the transmembrane protein 189 gene (*TMEM189*; *Tmem189* for the murine gene) encodes PEDS activity, that the conserved eight histidines found in a conserved motif occurring in TMEM189 proteins are essential for PEDS enzymatic activity, and that mice homozygous for an inactivated *Tmem189* gene have dramatically lower plasmalogen levels.

## Results

### Selection of Candidate Gene.

PEDS has never been purified but is thought to be a labile integral membrane protein (21). Therefore, we looked for a candidate gene to express or inactivate it in cultured cells and monitor the enzymatic activity using a fluorescence-based enzyme activity assay that we developed recently (22). In previous work, we found that the enzyme has properties similar to non–heme-containing di-iron desaturases (22), which are characterized by a motif of eight conserved histidines (23). The enzymatic activity was found in the microsomal fraction of tissues or cells (21, 22), thus being membrane-bound and likely originating from the endoplasmic reticulum. In our previous characterization of the gene for another ether lipid-metabolizing enzyme, alkylglycerol monooxygenase, we found good correlation of mRNA abundance with enzymatic activity in cells and tissues (24). Thus, for the selection of candidate genes, we followed the hypotheses that (*i*) the messenger RNA amount of the candidate would correlate with the enzymatic activity of the respective cell or tissue, (*ii*) the candidate should have features predicting it as a membrane protein, (*iii*) the candidate protein might have a kind of histidine motif characteristic for lipid desaturases, and (*iv*) the candidate gene should occur only in species synthetizing plasmalogens—that is, in animals but not in *Escherichia coli*, plants, yeast, or fungi.

In murine RAW264.7 cells, which have distinctive PEDS activity (22, 25) we found 9,619 of 24,421 genes clearly expressed by mRNA sequencing. We compiled these data with mRNA sequencing data obtained from the National Center for Biotechnology Information (NCBI) Gene Expression Omnibus (GEO) database for 11 human cell lines and seven mouse tissues. This resulted in 7,382 genes commonly expressed RAW 264.7 in 11 human cell lines and in seven mouse tissues. We then correlated for each of these genes, separately for human cell lines and for mouse tissues, the normalized mRNA sequencing counts with PEDS enzymatic activity that we had observed previously (22) (Fig. 1*B*). We ranked these 7,382 genes according to their mean correlation of cell and tissue mRNA sequencing counts with PEDS activity. *TMEM189* was the very top hit of all 7,382 genes examined, and both the gene symbol and the protein name defined it as a transmembrane protein. TMEM189 proteins contain a conserved motif (pfam10520) with eight conserved histidines (Fig. 1*C*). This motif had also been found in an alternative class of plant desaturases designated fatty acid desaturase type 4 (FAD4) (26). These desaturases introduce a *trans* double bond at position ∆3 into a hexadecanoyl residue at *sn*2 of phosphatidylglycerol in photosynthetic membranes of plants (26). The eight-histidine motif of pfam10520 resembles the classical eight-histidine stearoyl CoA desaturase motif (Fig. 1*D*) (23) but is different in the amino acid distances and the grouping of the histidines. In addition, one TMEM189 protein homolog per species was found throughout the animal kingdom, but not in *E. coli*, *Saccharomyces cerevisiae*, or *Arabidopsis thaliana*. Thus, the *TMEM189* gene best met all four of our criteria, and we tested this gene for its impact on PEDS activity.

### *TMEM189* Encodes for PEDS.

For the following experiments, we used different cell types chosen according to their suitability for the respective assays—for example, HEK293T for transfection of expression plasmids, A431 for siRNA experiments, and human haploid HAP1 (27) for generating a *TMEM189* knockout cell line. In HEK-293T cells, transfection of an expression plasmid for TMEM189 protein, but not for selected desaturases included as controls, resulted in significantly increased PEDS activity (*F*_(10,__33)_ = 6.801, *P* < 0.0001, one way ANOVA; *n* = 4) (Fig. 1*E*). Repetition of this experiment with C-terminally 6x myc-tagged proteins and investigation of protein expression by Western blot analysis with an anti myc-tag antibody confirmed expression of all recombinant proteins (*SI Appendix*, Fig. S1). Comparable to the results with the untagged proteins shown in Fig. 1*E*, also for the transfection of the myc-tagged versions of the proteins only TMEM189-6x myc led to a mean 2.37 ± 0.18-fold increase in PEDS activity (*n* = 4) compared with green fluorescent protein (GFP)-transfected cells. Knockdown of *TMEM189* mRNA by siRNA in A431 cells (Fig. 1*F*) led to significantly lower PEDS activity (*P* < 0.032, two-tailed, unpaired *t* test; *n* = 5).

We next obtained HAP1 cells with an inactivated *TMEM189* gene generated by specific gene editing with the CRISPR/Cas9 system. A 61-bp deletion of exon 5 resulted in a truncated protein lacking five of the eight conserved histidines of pfam10520 (*SI Appendix*). In contrast to wild-type (WT) HAP1 cells, *TMEM189*-deficient HAP1 cells had no detectable PEDS activity (Fig. 1*G*). Only an expression plasmid for TMEM189 protein, but not for other selected desaturase proteins, was able to restore PEDS activity in *TMEM189-*deficient HAP1 cells (Fig. 1*G*). Repetition of this experiment with C-terminally 6x myc-tagged proteins and investigation of protein expression by Western blot analysis with an anti-myc antibody confirmed expression of all recombinant proteins (*SI Appendix*, Fig. S1). Again, only TMEM189-6x myc yielded PEDS activity (mean, 0.39 ± 0.16 pmol mg^−1^ min^−1^; *n* = 3). *TMEM189-*deficient HAP1 cells had dramatically lower plasmalogen content compared with WT HAP1 cells (17-fold lower; *P* = 0.0015, two-tailed, unpaired *t* test) (Fig. 1*H*).

To measure de novo formation of the alk-1′-enyl ether double bond in intact cells, we fed WT HAP1 and *TMEM189-*deficient HAP1 cells for 24 h with 1*-O*-pyrenedecyl-*sn*-glycerol, which is taken up by cells and incorporated into plasmalogens (22). Owing to the accumulation of the metabolites in cells over 24 h, this gives a more sensitive readout than the enzymatic activity assay, in which the incubation time is limited to 30 min before the enzymatic activity levels off (22). Only an expression plasmid for TMEM189 protein, but not for selected other desaturase proteins, was able to yield the formation of pyrene-labeled plasmalogens. All other desaturase proteins tested did not catalyze formation of the alk-1′-enyl ether bond (<0.1% of TMEM189 transfection). Also with myc-tagged proteins, only transfection of TMEM189-6x myc resulted in the formation of alk-1′-enyl lipids (*SI Appendix*, Fig. S2), although all proteins were robustly expressed (*SI Appendix*, Fig. S1).

### *TMEM189*-Deficient HAP1 Cells Selectively Accumulate Plasmanylethanolamines.

**Fig. 2.**
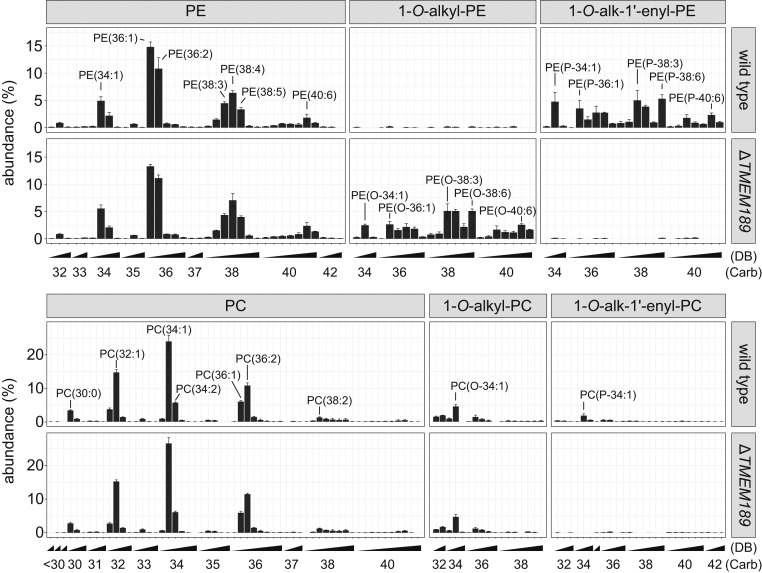
Glycerophosphoethanolamines and glycerophosphocholines in WT and *TMEM189*-deficient human HAP1 cells. *TMEM189*-deficient HAP1 cells (∆TMEM189) contain a frame-shift mutation introduced by a CRISPR-Cas9 protocol, leading to a truncated, inactive protein (*SI Appendix*). Cells were cultured under standard conditions, and lipids were extracted and analyzed by LC-MS/MS as described in *Materials and Methods*. Mean ± SD of three independent experiments is shown. PC, phosphatidylcholines (ester bond at *sn*1); PE, phosphatidylethanolamines (ester bond at *sn*1); 1-*O*-alkyl PC, plasmanylcholines [ether bond at *sn*1, PC(O−)]; 1-*O*-alk-1′-enyl PC, plasmenylcholines [vinyl ether bond at *sn*1, i.e., plasmalogens, choline type, PC(P-)] 1-*O*-alkyl PE, plasmanylethanolamines [ether bond at *sn*1, PE(O-)]; 1-*O*-alk-1′-enyl PE, plasmenylethanolamines [vinyl ether bond at *sn*1, i.e., plasmalogens, ethanolamine type, PE(P-)]. Numbers in parentheses give the total number of side chain carbon atoms (Carb), followed by the number of double bonds (DB). To facilitate location of species, selected bars are labeled.

An analysis of glycerophosphoethanolamines and glycerophosphocholines by liquid chromatography tandem-mass spectrometry (LC-MS/MS) revealed that plasmanylethanolamines, the substrates of PEDS, accumulated in *TMEM189*-deficient HAP1 cells displaying the same side chain pattern as the plasmenylethanolamines, which were found only in WT HAP1 cells (Fig. 2). Plasmenylcholines formed by the cells from plasmenylethanolamines (2) were less abundant and also absent in *TMEM189*-deficient cells. The ester-linked phosphatidylcholines and phosphatidylethanolamines, as well as the plasmanylcholine species, remained unchanged (Fig. 2).

### The Conserved Eight Histidines Are Absolutely Essential for PEDS Activity.

**Fig. 3.**
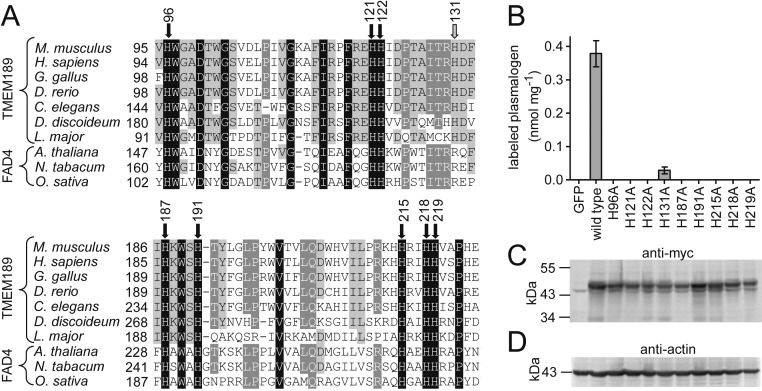
Importance of the eight-histidine motif for plasmalogen formation by transiently expressed TMEM189 protein. (*A*) Amino acid sequence comparison of pfam10520 motif containing proteins. Two regions containing the conserved histidines are shown. Black arrows indicate histidines conserved in all proteins; gray arrow, histidine conserved in TMEM189 proteins only. Numbers at arrows correspond to the position in the murine TMEM189 protein sequence. (*B*) Ability of plasmids expressing WT and mutated murine TMEM189 proteins to enable formation of pyrene-labeled plasmalogens on feeding with 1-*O*-pyrenedecyl-*sn* glycerol in *TMEM189*-deficient human HAP1 cells. At 24 h posttransfection with plasmids for TMEM189 proteins containing C-terminal 6x myc tags, cells were fed for another 24 h with 5 µM 1-*O*-pyrenedecyl-*sn*-glycerol. A plasmid for GFP served as a control. Values were related to the amount of cellular protein. Data are mean ± SEM of three experiments. (*C*) Expression of the recombinant proteins was monitored at 48 h posttransfection by Western blot analysis using an antibody against the C-terminal 6x myc tag. A representative example of three independent experiments is shown. (*D*) Western blots against β-actin to monitor cellular protein loading.

We next checked the importance of the eight conserved histidines found in the pfam10250 motif for PEDS enzymatic activity by site-directed mutagenesis to alanine. Fig. 3*A* shows an alignment of two conserved parts of TMEM189 proteins of mice, humans, and selected model organisms compared with three selected plant FAD4 proteins. In the murine TMEM189 protein, the eight histidines conserved in the pfam10250 motif carry numbers 96, 121, 122, 187, 191, 215, 218, and 219 (black arrows in Fig. 3*A*). In addition, we included histidine 131, which is conserved in all TMEM189 proteins but not in FAD4 plant desaturase proteins (gray arrow in Fig. 3*A*). We transfected expression plasmids carrying mutations of either of these histidines to *TMEM189*-deficient HAP1 cells and monitored the amount of pyrene-labeled plasmalogens formed on feeding of the transfected cells for 24 h with 1-*O*-pyrenedecyl-*sn*-glycerol. Pyrene-labeled plasmalogens were quantified in lipid extracts by HPLC with fluorescence detection of the amount of pyrenedecanal dimethylacetal formed from plasmalogens by treatment with HCl in methanol (Fig. 3*B*). Expression of the transfected proteins in the cells was confirmed by Western blot to the C-terminal 6x myc tag (Fig. 3*C*), with equal loading of cellular protein to all lanes by staining with β-actin (Fig. 3*D*). Each of the eight histidines conserved in pfam10250 was absolutely essential for PEDS activity as monitored by labeled plasmalogen formation in intact cells, whereas mutation of histidine 131 to alanine resulted in a strongly reduced but still detectable PEDS activity (Fig. 3*B*).

### A TMEM189-GFP Fusion Protein Localizes to the Endoplasmic Reticulum.

We next checked localization of the TMEM189 protein by expressing a TMEM189-GFP fusion protein in HEK293T cells and observed the localization of the fluorescent fusion protein by confocal microscopy. The expression pattern showed localization in the endoplasmic reticulum by an overlay of the green fluorescence signal with ER Tracker Red (*SI Appendix*, Fig. S3). Curiously, red fluorescence also appeared in vesicle-like structures that were devoid of GFP fluorescence. While mutation of the nine histidines to alanine strongly affected PEDS enzymatic activity (Fig. 3), localization of the GFP fusion proteins was not altered (*SI Appendix*, Fig. S3).

### *Tmem189*-Deficient Mice Lack Both PEDS Activity and Plasmalogens.

**Fig. 4.**
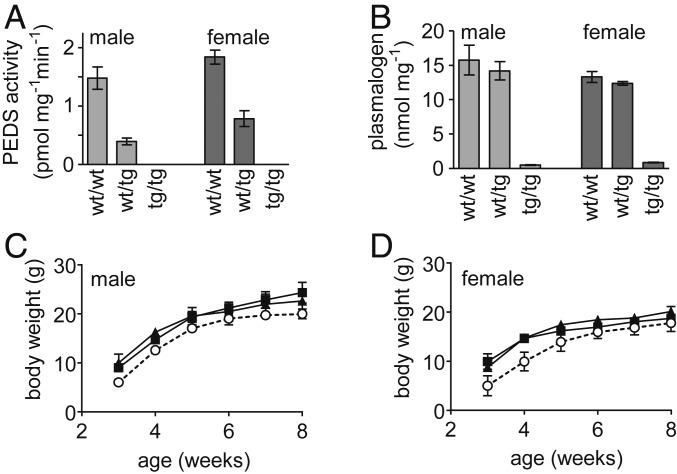
PEDS activities, plasmalogen levels, and body weight in mice depending on the *Tmem189* gene locus. Tmem189tm1a(KOMP)Wtsi mice were housed under standard conditions and weighed weekly, and kidneys were collected at 8 wk for PEDS activity and plasmalogen measurements as described in *Materials and Methods*. The tg was a knockout-first allele leading to an inactive truncated TMEM189 protein by artificially splicing a LacZ reporter downstream of exon 2 (28). (*A*) PEDS activity in kidney samples. (*B*) Plasmalogen levels in kidney samples. (*C*) Body weight in male animals. (*D*) Body weight in female animals. Open circles, dashed line: homozygous tg animals (tg/tg); solid squares, solid line: heterozygous animals (wt/tg); solid triangles, solid line: WT animals (wt/wt). Data are mean ± SEM for three animals each.

*Tmem189*-deficient mice were generated as part of the international mouse phenotype project (28) and subjected to a systematic phenotyping protocol (29). In these mice, we measured plasmalogen levels and PEDS activity in the kidneys, the organ with the highest PEDS activity (22). PEDS activities were diminished in heterozygotes compared with WT (two-tailed *P* = 0.0056 for males, 0.0044 for females, unpaired *t* test), and were below the detection limit of 0.2 pmol mg^−1^ min^−1^ (22) in animals homozygous for the transgene (tg) (Fig. 4*A*). In the tg construct, a galactosidase reporter is spliced downstream exon 2 to the TMEM189 protein, thus truncating the protein and inactivating the enzymatic activity (28). Plasmalogen levels remained largely unchanged by replacement of one WT allele with the inactive tg (two-tailed *P* = 0.577 for males and 0.333 for females; *n* = 3 each), whereas homozygous animals with both alleles as tg had almost completely lost their plasmalogens (two-tailed *P*, WT/WT vs tg/tg = 0.0021 for males, <0.0001 for females, unpaired *t* test; *n* = 3 each) (Fig. 4*B*). Body weight was reduced in homozygous male (*F*_(2,__6)_ = 9.305, *P* = 0.0145, two-way ANOVA; *n* = 3) (Fig. 4*C*) and female (*F*_(2,__6)_ = 9.653, *P* = 0.0133, two-way ANOVA; *n* = 3) (Fig. 4*D*) animals compared with heterozygous or WT animals. In addition to the kidneys (Fig. 4*B*), plasmalogens were also found to be strongly reduced in 12 other tissues of homozygous tg-carrying mice (*SI Appendix*, Fig. S4).

## Discussion

In this work, we show that the *TMEM189* gene is essential for the introduction of the alk-1′-enyl ether bond to form plasmalogens. TMEM189 proteins contain a conserved motif comprising eight conserved histidines that had previously been found in an alternative type of plant fatty acid desaturase (26). This motif differs in structure somewhat from the eight-histidine motif found in classical membrane bound desaturases, such as stearoyl CoA desaturase (23). The conserved histidines of stearoyl CoA desaturase have been shown to coordinate a di-metal center in crystal structures (30, 31) that is thought to be involved in catalysis of the enzymatic reaction. We found that each of the eight histidines conserved in both the TMEM189 proteins and the plant FAD4 alternative desaturase proteins is essential for PEDS activity. These data strongly suggest that the TMEM189 protein is the PEDS enzyme itself rather than an accessory protein required for the enzymatic reaction.

TMEM189 protein is currently annotated as an ubiquitin ligase in databases, and the pfam10520 protein motif is annotated as the localization B domain of TMEM189. This is based on findings that in human cells, a read-through transcript of *TMEM189* with the adjacent gene, the ubiquitin-conjugating enzyme E2 variant 1 (*UBE2V1*), was found (32). The authors characterized TMEM189 as a conserved class of proteins with a histidine-rich motif. When fused to the downstream gene *UBE2V1*, overexpression of the TMEM189 fusion protein altered localization of the UBE2V1 protein from the nucleus (32) to the endoplasmic reticulum (33), where unfused TMEM189 protein was also found (33). This gave rise to the designation of the pfam10520 domain as a localization domain. The read-through transcript was rare, however, and the two genes adjacent in humans were found to be separate in *Caenorhabditis elegans* as well as in *Drosophila melanogaster* (32), indicating that formation of the read-through transcript with UBE2V1 might not be the major role of TMEM189 proteins. Since we find here that the *TMEM189* encodes PEDS, we suggest naming the gene *PEDS* and the protein plasmanylethanolamine desaturase. Since the pfam10520 domain is also found in the FAD4 alternative type of plant fatty acid desaturase proteins, and the conserved histidines of this domain are essential for PEDS enzymatic activity, we further suggest annotating pfam10520 as a lipid desaturase domain.

We found that a mouse strain with inactivated *Tmem189* gene had no detectable PEDS activity in the kidneys, the organ with the otherwise highest PEDS activity (22). Plasmalogen levels were strongly reduced in all organs of the mouse that we tested, indicating that no isoenzyme encoded by a gene different from *Tmem189* is present in these tissues to carry out the PEDS reaction. Mice with inactivated *Tmem189* gene had significantly lower weight, indicating the importance of the vinyl ether bond for normal growth. The phenotype of *Tmem189*-deficient mice has already been characterized in a systematic phenotyping program (28), and results have been presented previously (29). Our finding of a lack of plasmalogens in these mice provides a novel biochemical basis for the interpretation of the phenotype. For example, plasmalogen deficiency accompanies diminished weight also in mouse strains with plasmalogen deficiency caused by inactivation of *Pex7*, *Gnpat*, or *Agps* genes, all of which are required for the peroxisomal steps of ether lipid biosynthesis (34). Growth retardation is also a hallmark of human peroxisomal ether lipid biosynthesis deficiency (10). In humans, a UK Biobank study found associations of single nucleotide polymorphisms in the *TMEM189* gene with altered monocyte percentage of white cells and with altered granulocyte percentage of myeloid white cells (35). However, alterations of monocyte or granulocyte cell counts were not evident in *Tmem189*-deficient mice (29).

*Pex7-*, *Gnpat-*, and *Agps-*deficient mice all show male infertility (34), whereas male *Tmem189*-deficient mice are fertile (29). This is consistent with the hypothesis that male infertility in mice with deficient peroxisomal ether lipid biosynthesis is caused by the lack of seminolipid (36), which has no vinyl ether bond and thus does not require PEDS to be synthesized. Interestingly, *Tmem189*-deficient mice have decreased bone mineral content (29). Humans with plasmalogen deficiency show a characteristic bone phenotype, rhizomelic chondrodysplasia punctata (10). For unknown reasons, this is observed mostly in proximal, but not in distal bone elements (34).

Mice with one WT allele contained >90% of plasmalogens of animals with both WT alleles, although the PEDS activity was even <50% of WT (Fig. 4). This indicates that PEDS might not be the rate-limiting step in plasmalogen tissue homeostasis, which is consistent with the suggestion that fatty acyl CoA reductase 1 controls the plasmalogen biosynthesis rate (37). In *TMEM189*-deficient human HAP-1 cells, we found accumulation of plasmanylethanolamines, the substrates of PEDS. The pattern of these plasmanylethanolamine species with regard to total carbon chain length and number of double bonds was strikingly similar to the pattern of plasmenylethanolamines, the products of the PEDS reaction, observed in WT cells. This shows that PEDS apparently does not discriminate between individual plasmanylethanolamine species for desaturation.

Our study provides a paradigm for the usefulness of shared public resources. High-throughput mRNA sequencing data from the NCBI GEO datasets for 11 human cell lines and seven mouse tissues allowed us to correlate gene expression with PEDS enzyme activity data that we had measured in our laboratory and directly led us to the gene for which we had searched. A mouse deficient in our gene of interest was available from the European Mutant Mouse Archive, and a systematic study of its phenotype had already been performed by the International Mouse Phenotyping Consortium, allowing us to interpret it on the basis of our biochemical findings regarding the role of the *Tmem189* gene in encoding PEDS. Our work will enable future investigations of specific roles of plasmalogens for mouse physiology by studying the *Tmem189*-deficient mouse in more detail with regard to known and assumed roles of this lipid class.

## Materials and Methods

More detailed information is provided in *SI Appendix*, *Materials and Methods*.

### High-Throughput mRNA Sequencing and Candidate Gene Selection.

mRNA sequencing of RAW264.7 cells was carried out using total RNA prepared in our laboratory. Further processing, including a PolyA enrichment step, was done by Microsynth. High-throughput mRNA sequencing data for seven mouse tissues and 11 human cell lines was downloaded from the GEO database (https://www.ncbi.nlm.nih.gov/gds/), and normalized to the same total read count. Mouse–human gene comparison was done by two independent methods, using data from the ensembl server (mmusculus(mm10/GRCm38.p1)) -hsapiens (hg19/GRCh37.p8, https://www.ensembl.org/) (38), and OrthoRetriever (https://lighthouse.ucsf.edu/orthoretriever/) (39). The results of the two combined mRNA seq datasets for 7,382 genes were then ranked according to their mean Pearson correlation, with PEDS activities calculated separately for the respective cells and tissues. The full dataset is available in *SI Appendix*. PEDS activity data were taken from our previous work (22) and are also available as a separate datafile in *SI Appendix*.

### Cultivation of Cells and Generation of a *TMEM189*-Deficient Human HAP1 Cell Line.

All cell lines were kept at 37 °C in a humidified atmosphere with 5% CO_2_ in media recommended by the suppliers containing 10% (vol/vol) FBS (F7524; Sigma-Aldrich), except that we used media free of antibiotics. Cells were from American Type Culture Collection. WT and *TMEM189*-deficient HAP1 cells were from Horizon.

### Transfection with Expression Plasmids and siRNAs.

Expression plasmids from Dharmacon or Origine and TurboFect (Thermo Fisher Scientific) or siGenome Smart pools from Dharmacon and ScreenFect were used. HAP1 cells were transfected with Turbofectin (Origene). Site-directed mutagenesis was performed with the Quikchange Kit (Stratagene).

### Real Time Live Confocal Microscopy.

Murine *Tmem189* cDNA was cloned into pEGF-N1 (Clontech), and the TMEM189-GFP fusion protein was expressed in HEK-293T cells. Real-time confocal imaging was performed at 48 h after transfection with a spinning-disk confocal system (UltraVIEW VoX; PerkinElmer) connected to a Zeiss AxioObserver Z1 microscope.

### Feeding of Cells with 1-*O*-pyrenedecyl-sn-Glycerol, Cell Harvest, and Lipid Extraction.

These steps were performed as described previously (22). 1-*O*-pyrenedecyl-*sn*-glycerol was obtained from Otava. Lipids were extracted from cell pellets twice with 500 µL of chloroform/methanol (2/1 vol/vol), and the combined organic phases were dried. The dried lipid extract was taken up in 100 µL of acetonitrile/ethanol (1/1 vol/vol) and stored at −20 °C until analysis.

### Measurement of PEDS Enzymatic Activity.

PEDS activity assays were performed as described previously (22). The fluorescent 1-*O*-pyrenedecyl-*sn*-glycero-3-phosphoethanolamine substrate was purified from lipid extracts of 1-*O*-pyrenedecyl-*sn*-glycerol–treated RAW.12 cells (25) with a protocol comprising cleavage of residual plasmenyl species by HCl, a first reversed-phase HPLC purification step, cleavage of the 2-acyl side chains by NaOH, and then a second HPLC purification step (22). After incubation with NADPH and catalase, the reaction was stopped with acetonitrile/HCl to liberate pyrenedecanal from the plasmalogen formed, which was quantified by reversed-phase HPLC and fluorescence detection. Controls with acetic acid/acetonitrile to quantify non–plasmalogen-derived pyrenedecanal were always negative.

### Measurement of Labeled Alkyl and Alk-1′-Enyl Lipids.

Pyrene-labeled alkyl and alk-1′-enyl lipids were quantified by reversed-phase HPLC with fluorescence detection as described (22), with the modification that methanol rather than acetonitrile was used together with HCl (or acetic acid as a control) to liberate pyrenedecanal from plasmalogens, and that the aldehyde was quantified as the resulting pyrenedecanal dimethylacetal.

### Measurement of Total Unlabeled Plasmalogen.

This step was performed as described previously (22). Lipid extracts were derivatized with dansylhydrazine (Sigma-Aldrich) in acetonitrile in the presence of either HCl (to quantify plasmalogens plus free aldehydes) or acetic acid (to quantify free aldehydes only, which were typically <1% of plasmalogens). The resulting dansylhydrazones were determined by reversed-phase HPLC with fluorescence detection (22).

### LC-MS/MS of Glycerophosphocholines and Glycerophosphoethanolamines.

This step was performed as described previously (40), modified and extended to also allow quantification and fragmentation of glycerophosphocholines and glycerophosphoethanolamines. Internal standards were added to cell homogenates, and lipids were extracted via the Folch procedure (41). Dried lipid extracts were dissolved in HPLC starting condition, separated by reversed-phase HPLC on a Dionex Ultimate 3000 HPLC (Thermo Fisher Scientific), and quantified with a Velos Pro Dual-Pressure Linear Ion Trap Mass Spectrometer (Thermo Fisher Scientific). Baseline corrected data were integrated in MZmine 2 (42), quantified, and visualized as described previously (40) using custom-made scripts in R (https://www.R-project.org/). Molecular PE, PC, plasmanyl-PE and plasmenyl-PE species were identified by their retention time, monoisotopic mass-to-charge ratio, isotope pattern, and fragmentation behavior (*SI Appendix*, Fig. S5 and *Materials and Methods*), which was cross-validated with single lipid standards commercially available from Avanti Polar Lipids: C16-18:1 PC, C18(Plasm)-22:6 PC, C16-18:1 PE, C18(Plasm)-18:1 PC, C18(Plasm)-18:1 PE, and C18(Plasm)-20:4 PC.

### Western Blot Analysis.

This analysis was performed using standard techniques and antibodies against the myc tag (ab 9106, Abcam) and β-actin (MAB1501; Merck Millipore), Cy3- and Cy5-labeled secondary antibodies (GE Healthcare), and a Typhoon 9410 multi-wavelength laser scanner (GE Healthcare).

### Harvest of Mouse Tissues for Analysis of PEDS Activity and Plasmalogen Content.

Animal breeding practices were approved by the Austrian Ministry of Education, Science, and Culture (BMBWF-66.011/0100-V/3b/2019). Tmem189tm1a(KOMP)Wtsi mice (Welcome Sanger Institute) were maintained on C57bl/6N genetic background. The tg was a knockout-first allele leading to an inactive truncated TMEM189 protein by artificially splicing a galactosidase reporter downstream of exon 2 (28). The mice were housed in individual ventilated cages with nesting material, on a 12-h/12-h light/dark cycle with standard chow and water ad libitum. Mice were weighed weekly from 3 to 8 wk of age. For tissue harvest, 8-wk-old female and male homozygous *Tmem189*-deficient mice and their heterozygous and WT littermates were euthanized by cervical dislocation. Tissues were snap-frozen in liquid nitrogen and stored at −80 °C until further analysis.

### Data Availability.

The complete dataset of compiled RNA sequencing of RAW264.7, seven mouse tissues, and 11 human cell lines and their correlation to PEDS activity, as well as the PEDS activity data used to calculate the correlation, are available as data files in *SI Appendix*.

### Note Added in Proof.

A recent study characterizing a bacterial light response also found that the *TMEM189* gene encodes PEDS (44).

## Supplementary Material

Supplementary File

Supplementary File

Supplementary File
