# Supplementary material for: The TMEM189 gene encodes plasmanylethanolamine desaturase which introduces the characteristic vinyl ether double bond into plasmalogens
Source: Proc Natl Acad Sci U S A. 2020 Mar 24;117(14):7792–8. doi: 10.1073/pnas.1917461117 (PMC7149458; doi:10.1073/pnas.1917461117)
Supplement: Supplementary File [file pnas.1917461117.sapp.pdf]

Supplementary Information for

The TMEM189 gene encodes plasmanylethanolamine desaturase which introduces the characteristic vinyl ether double bond into plasmalogens

Ernst R. Werner, Markus A. Keller, Sabrina Sailer, Katharina Lackner, Jakob Koch, Martin Hermann, Stefan Coassin, Georg Golderer, Gabriele Werner-Felmayer, Raphael A Zoeller, Nicolas Hulo, Johannes Berger, Katrin Watschinger

Ernst R. Werner, Katrin Watschinger
Email: ernst.r.werner@i-med.ac.at, katrin.watschinger@i-med.ac.at

This PDF file includes:

Supplementary text
Figures S1 to S5
SI References

www.pnas.org/cgi/doi/10.1073/pnas.1917461117

Supplementary Information Text
Materials and Methods
mRNAseq evaluation and candidate gene selection

Total RNA was prepared from RAW264.7 cells (ATCC, Manassas, VA, USA) using the Qiagen RNA isolation kit (Qiagen, Hilden, Germany) with DNAse treatment for five independent cultures. This was shipped to Microsynth (Balgach, Switzerland), where a stranded TrueSeq RNA library (Illumina, Cambridge, UK) was prepared including a polyA enrichment step. Sequencing was done with an Illumina NextSeq,v2, 1 x 75 bp (360 million reads post filtering, mean read length 75 bp). After demultiplexing and trimming of Illumina adaptor residuals, reads were mapped to the mouse reference genome (*Mus musculus*, mm10) using STAR (v.2.5.1b). Mapped reads were counted with HTSeq (v 0.6.0). Compilation of RNAseq data was done by Microsoft Excel 2010 and by R (versions 3.2.2 and 3.5.2; (R Core Team (2019). R: A language and environment for statistical computing. R Foundation for Statistical Computing, Vienna, Austria. URL https://www.R-project.org/)). Starting from 9619 mouse genes expressed above threshold (> 100 normalized reads) detected in RAW264.7, which had a distinctive PEDS activity (1), a data file containing reads of 7 mouse tissues and 11 human cells lines was compiled by the use of data from NCBI GEO data sets (GSE74747 for mouse kidney, liver, lung spleen, testis, heart and brain; GSE94497 for MCF-7; GSE80082 for A431; GSE90322 for HepG2; GSE89431 for SK-N-SH and SH-Y-5Y; GSE43925 for THP-1; GSE97768 for T24; GSE72577 for DLD-1; GSE103706 for HL-60; GSE96649 for A549; GSE90830 for CaCo-2). Counts were normalized to the same total reads (65397515), and Pearson correlations calculated between reads and PEDS activity data (1) separately for the 7 mouse tissues and the 11 human cell lines. Mouse – human gene compilation was done by two methods, using data from the ensembl server (mmusculus(mm10/GRCm38.p1) – hsapiens (hg19/GRCh37.p8, https://www.ensembl.org/biomart/), and OrthoRetriever (https://lighthouse.ucsf.edu/orthoretriever/), both accessed June 28, 2018, and the results of the two combined. This resulted in 7382 of the 9619 genes expressed in RAW264.7 with enough data to compute correlations with PEDS activity for both mouse tissues and human cell lines.

Protein sequence comparison

This was done with the ClustalW package incorporated into the Mega6 program package (2) using the identity option for the protein weight matrix. Shading of the stapled proteins was done with GeneDoc with inactivated similarity shading option (GeneDoc version 2.7.000, Nicholas Karl B. and Nicholas, Hugh B., Jr, 1997, GeneDoc, a tool for editing and annotating multiple sequence alignments. Distributed by the author). TMEM189 proteins from selected organisms were compared to three selected FAD4 alternative desaturase proteins from plants. Accession numbers of protein sequences used were: TMEM189, *Mus musculus*, NP_663513.1; *Homo sapiens*, NP_954580.1; *Gallus gallus*, NP_001192029.1; *Danio rerio*, NP_001006052.1; *Caenorhabditis elegans*, NP_493036.1; *Dictyostelium discoideum*, XP_646227.1; *Leishmania major*, XP_001682977.1; FAD4 *Arabidopsis thaliana*, NP_194433.1; *Nicotiana tabacum*, XP_016466208.1; *Oryza sativa* , XP_015648482.1.

Cultivation of cells

All cell lines were kept at 37°C in a humidified atmosphere with 5% CO_2 in media recommended by the suppliers containing 10% (v/v) fetal bovine serum (F7524, Sigma, Vienna, Austria) except that we used media free of penicillin and streptomycin. Cells were from American type culture collection (Manassas, VA, USA) except for wild type and TMEM189 deficient HAP1 which were from Horizon (Cambridge, UK). Media employed were very low endotoxin DMEM (FG1445, Biochrom, Berlin, Germany) for A431, HEK293T, HEP-G2 and RAW264.7 and IMDM (GIBCO 12440-053 obtained via Fisher Scientific, Vienna, Austria) for HAP1.

Generation of a *TMEM189* deficient human HAP1 cell line

TMEM189 deficient cells were generated using the near haploid human HAP1 cell line (3) by Horizon (Cambridge, UK) upon our request. AGTGGTCGCACACGTACTTT was used as guide RNA sequence. The cell clone we obtained had a 61 bp deletion in exon 5 resulting in a frame shift and an early stop. This deleted five of the eight histidines of the TMEM189 protein which are conserved in pfam 10520, see details below.

LOCUS   huTMEM189   752 bp   DNA
DEFINITION cds of mRNA in HAP_1 CRISPR ko line (deduced from genomic sequence; break point with inserted T indicated in bold underlined).
ORIGIN
1 ATGGCGGGCG CCGAGGACTG GCCGGGCCAG CAGCTGGAGC TGGACGAGGA CGAGGCGTCT
61 TGTTGCCGCT GGGGCGCGCA GCACGCCGGG GCCCGCGAGC TGGCTGCGCT CTACTCGCCA
121 GGCAAGCGCC TCCAGGAGTG GTGCTCTGTG ATCCTGTGCT TCAGCCTCAT CGCCCACAAC
181 CTGGTCCATC TCCTGCTGCT GGCCCGCTGG GAGGACACAC CCCTCGTCAT ACTCGGTGTT
241 GTTGCAGGGG CTCTCATTGC TGACTTCTTG TCTGGCCTGG TACACTGGGG TGCTGACACA
301 TGGGGCTCTG TGGAGCTGCC CATTGTGGGG AAGGCTTTCA TCCGACCCTT CCGGGAGCAC
361 CACATTGACC CGACAGCTAT CACACGGCAC GACTTCATCG AGACCAACGG GGACAACTGC
421 CTGGTGACAC TGCTGCCGCT GCTAAACATG GCCTACAAGT TCCGCACCCA CAGCCCTGAA
481 GCCCTGGAGC AGCTATACCC CTGGGAGTGC TTCGTCTTCT GCCTGATCAT CTTCGGCACC
541 TT**T**TGCAGGA CTGGCATGTC ATCCTGCCAC GTAAACACCA TCGCATCCAC CACGTCTCAC
601 CCCACGAGAC CTACTTCTGC ATCACCACAG GCTGGCTCAA CTACCCTCTG GAGAAGATAG
661 GCTTCTGGCG ACGCCTGGAG GACCTCATCC AGGGCCTGAC GGGCGAGAAG CCTCGGGCAG
721 ATGACATGAA ATGGGCCCAG AAGATCAAAT AA
//
LOCUS   huTMEM189   219 aa
DEFINITION protein deduced from genomic sequence of HAP_1 CRISPR ko line. (histidines conserved in pfam 10520 bold underlined; amino acids differing from wild type in italic dotted underlined)
ORIGIN
1 MAGAEDWPGQ QLELDEDEAS CCRWGAQHAG ARELAALYSP GKRLQEWCSV ILCFSLIAHN
61 LVHLLLLARW EDTPLVILGV VAGALIADFL SGLV**H**WGADT WGSVELPIVG KAFIRPFRE**H**
121 **H**IDPTAITRH DFIETNGDNC LVTLLPLLNM AYKFRTHSPE ALEQLYPWEC FVFCLIIFGT
181 F*CRTGMSSCH VNTIASTTSH PTRPTSASPQ AGSTTLWRR**

Transfection with expression plasmids and siRNAs

HEK293T cells were seeded at a density of 5 x 10^5 in 6 well plates and transfected on the next day with 4 µg of expression plasmid and 6 µl TurboFect (Thermo-Fisher, Vienna Austria) in a total volume of 4 ml. Mammalian expression plasmids (CMV promoter) were from the Mammalian Genome Collection (for the murine proteins DEGS1, DEGS2, FADS1, FADS2, FADS6, SCD1, SCD2, TMEM189, Dharmacon, Lafayette, CO, USA) or from Origene (for the human proteins FADS3, FA2H, Origene, Rockville, MD USA). Human or murine sequences were chosen according to availability of error-free clones suited directly for mammalian expression as well as according to pricing. Untagged clones were used to avoid potential inactivation of the enzymatic activity by the tag, C-terminally 6xmyc-tagged clones were prepared from these to enable monitoring of recombinant protein formation by Western blot. Site directed mutagenesis was performed with the Quikchange Kit (Stratagene, La Jolla, CA, USA). Clones were regrown, purified with an endotoxin-free plasmid preparation protocol with the endo-free Kit (Qiagen) and sequenced (Microsynth). HAP1 cells were seeded at a density of 8 x 10^5 in 6 well plates and transfected with 1 µg DNA and 5 µl TurboFectin (Origene) in a total volume of 2 ml. A431 cells were seeded at a density of 3 x 10^5 and treated with 25 pmol siGenome Smart pools (Dharmacon) and 4 µl ScreenFect siRNA reagent (ScreenFect, Eggenstein-Leopoldshafen, Germany) in a total volume of 1.5 ml.

Real time live confocal microscopy

Murine *Tmem189* cDNA was cloned into pEGF-N1 (Clontech, Mountain View, CA, USA), the individual histidine to alanine mutations inserted using the Quikchange Kit (Stratagene) and the plasmids transfected to HEK293T cells which were seeded in a concentration of 5 x 10^4 cells/well in 8-well chambered cover glass slides (Ibidi µ-slide 8 well™ slides, Ibidi, Munich, Germany). Real time confocal imaging was performed 48 hours after transfection with a spinning disk confocal system (UltraVIEW VoX; Perkin Elmer, Waltham, MA) connected to a Zeiss AxioObserver Z1 microscope (Zeiss, Oberkochen, Germany). GFP-constructs were excited with a wavelength of 488 nm. Hoechst 33342 (Thermo Fisher Scientific, Waltham, MA, USA) was excited with 405 nm. ER Tracker Red (Thermo-Fisher) was added 100 nM to the cells and excited with 561 nm. Images

were acquired with the Volocity software (Perkin Elmer) using a 40 x water immersion objective with a numerical aperture of 1.2.

Feeding of cells with 1-O-pyrenedecyl-sn-glycerol
To monitor the formation of labeled plasmalogens in intact cells, cells were fed with 5 µM 1-*O*-pyrenedecyl-*sn*-glycerol (Otava, Vaughan, Ontario, Canada) which was first dissolved 4 mM in ethanol, diluted 100 fold with culture medium and then added to the cells.

Cell harvest and lipid extraction
Cells were collected by centrifugation, washed with phosphate-buffered saline, the phosphate-buffered saline aspirated, the pellets shock-frozen in liquid nitrogen and stored at -80°C until analyzed for PEDS activity, lipid content, total protein amounts and amounts of 6xmyc-tagged protein or ß-actin by Western blot. Lipids were extracted twice with 500 µl chloroform/methanol (2/1 v/v) and the combined organic phases dried. The dried lipid extract was taken up in 100 µl acetonitrile/ethanol (1/1 v/v) and stored at -20°C until analyzed.

Measurement of PEDS enzymatic activity
PEDS activity assays were performed with cellular homogenates using the fluorescent 1-*O*-pyrenedecyl-*sn*-glycero-3-phosphoethanolamine as substrate which was purified from lipid extracts of 1-*O*-pyrenedecyl-*sn*-glycerol treated RAW.12 cells as described(1). Briefly, extracts were treated with HCl to cleave residual alk-1'-enyl compounds, purified by a first reversed phase HPLC step, then the 2-acyl residue cleaved off by NaOH treatment, and the lyso compound isolated from a second reversed phase HPLC step, the solvent removed by evaporation, stored at -20°C and aliquots reconstituted with methanol to a concentration of 100 µM. Cellular pellets were homogenized in 200 µl buffer containing 0.1 M Tris and 0.25 M sucrose, pH 7.6 (HCl) using glass beads and four cycles of 30 s and 20 Hz in a Retsch MM400 homogenizer (Retsch, Haan, Germany). To ensure measurements in the linear range of the assay, protein was then determined by a Bradford assay (Biorad, Vienna, Austria) and the homogenates diluted with 3.5 mg/ml bovine serum albumin in phosphate buffered saline to yield cellular protein of 0.5 mg/ml (for expression plasmid testing) or 1 mg/ml (for si-RNA and mouse tissue testing). 7.5 µl of the diluted homogenates were mixed with 5 µl assay mix to start the reaction. The assay mix contained the following components (2.5 fold of the final assay concentrations); 0.25 M Tris.HCl, pH 7.2, 0.25 mg/ml catalase (C1345, Sigma), 2.5 mM NADPH, 5 mM EDTA, 5 µM 1-*O*-pyrenedecyl-*sn*-glycero-3-phosphoethanolamine (added from a 100 µM stock in methanol). After 30 min at 37°C, the reaction was stopped by adding 37.5 µl HCl/acetonitrile (105 parts 2 M aqueous HCl and 895 parts acetonitrile v/v), incubated for 30 min at 37°C, centrifuged for 5 min at 20000 g and 4°C, and 10 µl injected to an Agilent 1200 HPLC system (Agilent, Vienna, Austria). A Zorbax Eclipse XDB C8 column (4.6mm x 50mm, 3.5 µm particle size, Agilent) thermostatted to 25°C was eluted with a flow rate of 1 ml/min with 10 mM potassium phosphate buffer, pH 6.0, containing 79% (v/v) methanol for 3 min followed by a linear gradient to 100% methanol at 10 min. Methanol (100%) was held until 15 min, changed with a linear gradient to starting buffer until 15.5 min, which was held to 17 min. Pyrene labeled compounds were detected by fluorescence (340 nm excitation, 405 nm emission) and quantified by comparing peak areas in relation to external standards of pyrenedecanoic acid (Sigma). Retention time of the product pyrenedecanal was calibrated with external synthetic standard (Ramidus, Lund, Sweden). Pyrenedecanal was not used for quantification of the amounts by area comparison since it was less stable as pyrenedecanoic acid. Parallel incubations were stopped with acetonitrile containing the same amount of aqueous acetic acid instead of HCl to control for free pyrenedecanal formed in the incubations. Pyrenedecanal in these incubations was always below the detection limit, indicating all pyrenedecanal observed in the incubation mixtures originated from alk-1'-enyl compounds.

Measurement of labeled alkyl and alk-1'-enyl lipids
Pyrene labeled alkyl and alk-1'-enyl lipids were quantified by reversed phase HPLC with fluorescence detection with the same reversed phase HPLC system as described above for PEDS activity determinations, but with a slightly modified elution protocol. The column was eluted with 10 mM potassium phosphate buffer, pH 6.0, containing 81.25% (v/v) methanol for 5 min followed by a

linear gradient to 100% methanol at 10 min. Methanol (100%) was held until 21 min, changed with a linear gradient to starting buffer until 21.5 min, which was held to 23 min. Pyrene labeled compounds were detected by fluorescence (340 nm excitation, 405 nm emission) and quantified by comparing peak areas in relation to external standards of pyrenedecanoic acid (Sigma). To determine the amount of pyrene-labeled alk-1-'enyl lipids, lipid extracts were treated with HCl which cleaves the alk-1'-enyl ether bond to the respective aldehyde. Controls were treated with acetic acid which leads to the same aldehyde derivatives as HCl but leaves the alk-1'-enyl ether bond intact. These control incubations would result in the detection of free pyrenedecanal in the cell extracts which was, however, never observed. In a variation of our described protocol (1) we treated the extracts with HCl or acetic acid in methanol rather than in acetonitrile to monitor the liberation of pyrenedecanal from pyrene-labeled alk-1'-enyl lipids. Under these conditions, pyrenedecanal was converted to pyrenedecanal dimethylacetal which was better resolved as the free pyrenedecanal from the peak of 1-*O*-pyrenedecyl-*sn*-glycerol used to feed the cells. 10 µl lipid extract was mixed with 40 µl HCl/methanol (70 parts 2 M aqueous HCl/930 parts methanol v/v) or with 40 µl acetic acid/methanol (70 parts 2 M aqueous acetic acid/930 parts methanol v/v) and incubated for 30 min at 37°C to ensure complete cleavage and derivatization. The retention times of the pyrenedecanal derivatives as well as their formation under the indicated conditions were evaluated using synthetic pyrenedecanal (Ramidus AB, Lund, Sweden).

Measurement of total unlabeled plasmalogen

This was performed as described (1). Briefly, 10 µl lipid extracts were derivatized with 40 µl 0.45 mg/ml dansylhydrazine (Sigma) in acetonitrile in presence of either HCl (105 parts 2 M aqueous HCl/895 parts acetonitrile v/v) or acetic acid (105 parts 2 M aqueous acetic acid/895 parts acetonitrile v/v). The resulting aldehyde derivatives were separated with the same chromatographic method as described above for the determination of PEDS enzymatic activity except for the detection by fluorescence at excitation 340 nm, emission 525 nm. The method was calibrated with external standards 1-(1Z-octadecenyl)-2-oleoyl-*sn*-glycero-3-phosphoethanolamine, Avanti Polar Lipids, Alabaster, AL, USA) and hexadecanal which had been synthesized as described in (4). The amount of free aldehydes measured by incubations with acetic acid was subtracted from the amount of plasmalogens determined by incubations with HCl. The amount of free aldehydes was, however, typically below 1% of the measured amount of plasmalogens.

LC-MS/MS of glycerophosphocholines and glycerophosphoethanolamines

Analysis was performed similarly to (5) in a modified and extended version allowing quantification and fragmentational elucidation of glycerophosphocholines and glycerophosphoethanolamines. Briefly, the harvested cell pellets (approximately 1 million cells) were stored at -80°C prior to Folch lipid extraction (6), at the beginning of which 5 µM PC (28:0) and PE (28:0) were added as internal standards. Cells were broken in 200 µl deionized water with the help of glass beads (Sigma) and a 10 µl aliquot of the aqueous sample was removed prior to lipid extraction for protein content measurement by a Bradford assay (Biorad, Vienna, Austria) with bovine serum albumin as standard. Samples were then shaken, sonicated on ice, centrifuged at 20000 x g and 4°C, and the lower organic residue transferred to a new glass vial and extraction was repeated without sonication. For LC-MS/MS measurements the extracts were dissolved in HPLC starting solvent (54% A, 46% B (v/v), adjusted by protein content and 10 µl separated by reversed-phase HPLC using a Poroshell 120 EC-C8 2.7 µm 2.1 x 100 mm column (Agilent). Solvent A consisted of 10 mM ammonium formate, 0.2% formic acid in isopropanol/acetonitrile (9/1 v/v). Solvent B consisted of 10 mM ammonium formate, 0.2% (v/v) formic acid in acetonitrile/water 6/4 (v/v). At a flow rate of 0.4 ml/min, the column was first eluted for 2 min with 54% A, 46% B (v/v) followed by a linear gradient to 28% A, 72% B (v/v) at 22 min. After rinsing with 100% B and 0.6 ml/min from 23 to 28 min, the column was re-equilibrated for another 2 min at starting conditions before the next injection. A Dionex Ultimate 3000 HPLC (Thermo Fisher Scientific) coupled to a Velos Pro Dual-Pressure Linear Ion Trap Mass Spectrometer (Thermo Fisher Scientific) was employed. A dilution series of (28:0) and (36:2) PC and PE standards was measured at the beginning and end of the batch. 5 µl of each sample were pooled as quality control sample measured before and after the samples. The samples were measured consecutively in randomized order. Data analysis was performed in Mzmine2 (7), Version 2.40 applying an in-house built custom database paired with

manual correction and verification on baseline corrected data. Quantification was performed by external calibration using our in-house R data analysis workflow. For data analysis class- wise normalized profiles were used for mean and SD calculation and plotting. Only a handful of different 1-*O*-alkyl (plasmanyl) lipids and 1-*O*-alk-1'enyl (plasmenyl) lipids are commercially available, therefore their behavior (relative to class specific standards and other phospholipids) was extrapolated. Briefly, we found for isobaric 1-*O*-alkyl (plasmanyl) lipids and 1-*O*-alk-1'-enyl (plasmenyl) lipids (for PE as well as PC) that the 1-*O*-alkyl (plasmanyl) lipids have a 45-60 s shorter retention time on our reversed-phase HPLC system as compared to their isobaric plasmenyl (1-*O*-alk-1'enyl) counterparts. This was matched by the respective MS/MS fragmentation behavior. With our ESI ion source and CID fragmentation, plasmanyl as well as plasmenyl lipids fragment at the *sn*2 fatty acyl residue, yielding a mass for the *sn*2-acyl residue and the complementary headgroup-1-*O*-alkyl/alk-1'-enyl residue. Together with the precursor mass and retention time, this constrains the possible molecular structure down to the fatty acyl level, but does for example not include information about the double bond position on the *sn*2 residue. See Supplementary Figure 5 for an example for retention times and the fragmentation spectra for selected plasmanyl- and plasmenyl lipid species.

Western blot

Cells were homogenized as described above for the PEDS enzymatic assay, protein determined by a Bradford assay (Biorad) and 60 µg cellular protein heated for 5 min at 95°C by addition of 1/5 (v/v) 5x sample buffer (60 mM Tris.HCl, pH 6.8, 25% v/v glycerin, 2% sodium dodecyl sulfate, 0.1% (w/v) bromophenol blue, 5% (v/v) mercaptoethanol), centrifuged for 10 min at 100000 g and 25°C in a table top ultracentrifuge (Sorvall MTX 150, Thermo Scientific, Vienna, Austria) and separated on polyacrylamide gels (10 cm long, 18 cm wide, 1.5 mm think) containing 10% sodium dodecyl sulfate. Proteins were blotted onto polyvinylidene difluoride membranes (GE Healthcare, Vienna, Austria), blocked for 2 h at room temperature with 3% bovine serum albumin in phosphate buffered saline containing 0.1% (v/v) Tween 20, (Serva, Heidelberg, Germany). 6xMyc tags were detected with a rabbit polyclonal anti-myc tag antibody (ab 9106, abcam, Milton, UK) diluted 1:5000 (v/v) in blocking solution incubated together with a mouse monoclonal pan actin antibody (MAB1501, Merck Millipore, Vienna, Austria) 1:2000 (v/v) diluted in the blocking solution. After washing with 0.1% Tween 20 in phosphate buffered saline, myc tags were stained with a goat anti rabbit Cy5 antibody (1:1250; GE Healthcare), scanned on a Typhoon 9410 fluorescence scanner (GE Healthcare, excitation 633 nm, emission 670 nm). ß-Actin was subsequently visualized by incubation with a goat anti mouse Cy3 antibody (1:1250; GE Healthcare), and scanned at excitation 532 nm, emission 580 nm.

Harvest of mouse tissues for analysis of PEDS activity and plasmalogen content

Animal breeding was approved by the Austrian Ministry of Education, Science, and Culture (BMBWF-66.011/0100-V/3b/2019). Tmem189tm1a(KOMP)Wtsi mice were obtained from the Welcome Sanger Institute (Hinxton, Cambridge, UK) and were maintained on C57bl/6N genetic background. The transgene (tg) was a knockout-first allele leading to an inactive truncated Tmem189 protein by artificially splicing a galactosidase reporter downstream of exon 2 (8). Mice were housed in individual ventilated cages with nesting material, in a 12 h/12 h light/dark cycle with standard chow and water ad libitum. Genotyping was performed as recommended by the supplier, with primers Tmem189_35635F (GCGTGTCCTGCTGAGACTTG) and CAS_R1_Term (TCGTGGTATCGTTATGCGCC) for the transgenic allele and Tmem189_35635F and Tmem189-35635R (CATCCCACCTATCCCACCTG) for wildtype allele. Mice were weighed weekly from 3-8 weeks of age. For tissue harvest 8 week old female and male homozygous Tmem189 deficient mice and their heterozygous and wild type littermates were sacrificed by cervical dislocation. Tissues were snap frozen in liquid nitrogen and stored at -80°C until further analysis.

Statistical calculations

Statistical calculations were performed with PRISM (version 8) GraphPad software, San Diego, CA. USA.

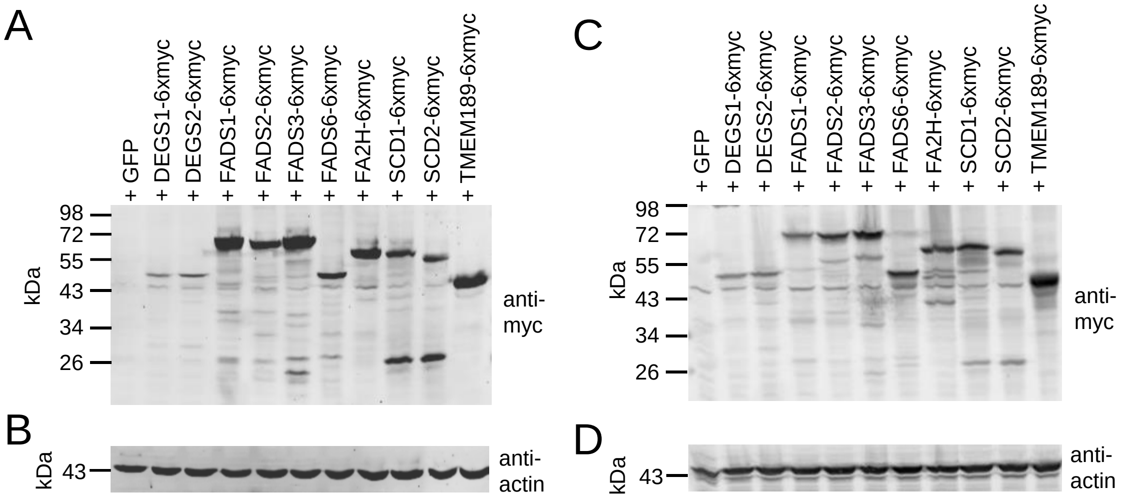

Fig. S1. Expression of recombinant desaturase and hydroxylase proteins in cells as assessed by Western blot

(A) HEK293T cells were transfected with the indicated expression plasmids of C-terminally 6xmyc tagged proteins, cells collected 48 h post transfection and analyzed by Western blot using an anti-myc antibody. *(B)* Staining of the blot shown in *(A)* with an antibody to ß-actin to demonstrate equal protein loading. *(C) TMEM189*-deficient HAP1 cells were transfected with the indicated expression plasmids of C-terminally 6xmyc tagged proteins, cells collected 48 h post transfection and analyzed by Western blot using an anti-myc antibody. *(D)* Staining of the blot shown in *(C)* with an antibody to ß-actin to demonstrate equal protein loading. Calculated molecular masses (kDa) of the fusion proteins are DEGS1-6xmyc, 50.4; DEGS2-6xmyc, 49.5; FADS1-6xmyc, 65.3; FADS2-6xmyc, 65.1; FADS3-6xmyc, 64.5; FADS6-6xmyc, 51.7; FA2H-6xmyc, 56.3; SCD1-6xmyc, 53.6; SCD2-6xmyc, 53.4; TMEM189-6xmyc, 43.9. One representative example of three independent experiments is shown.

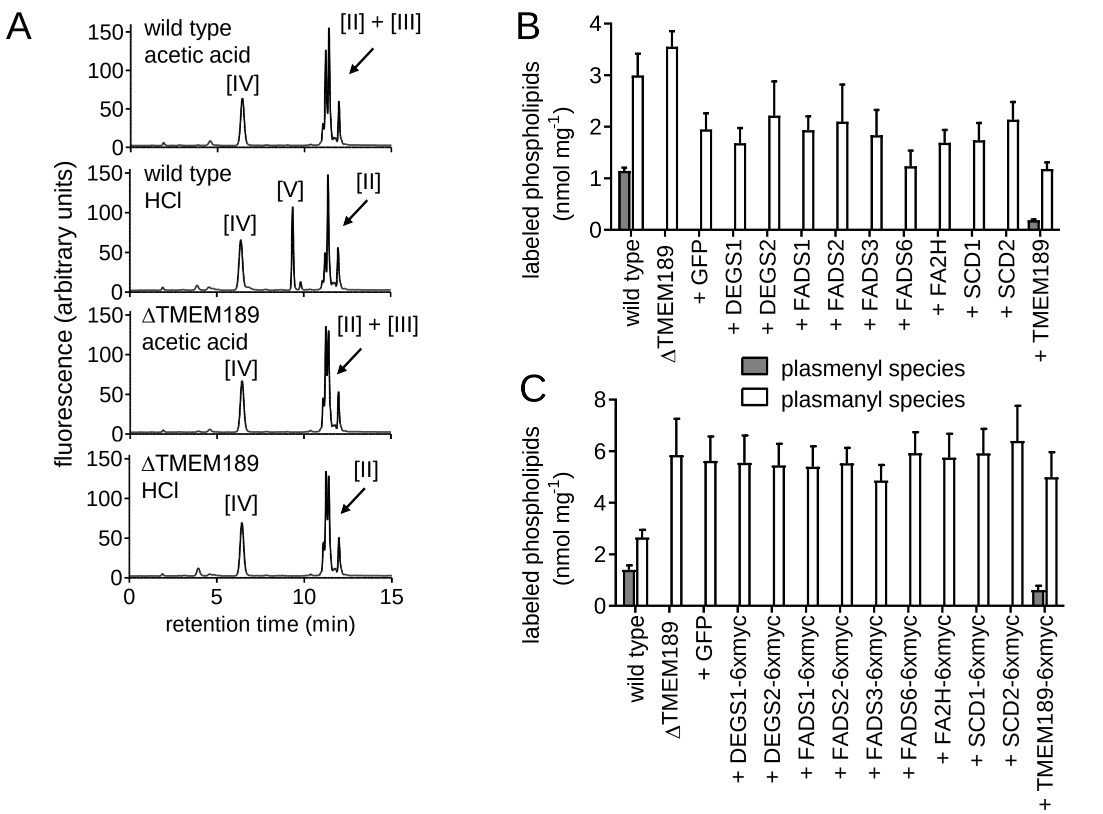

Fig. S2. Synthesis of plasmalogens from fed pyrene labeled alkylglycerol in intact cells. *(A)* Reversed phase HPLC chromatograms of lipid extracts of wild type and *TMEM18*9-deficient (ΔTMEM189) HAP1 cells fed with 1-*O*-pyrenedecyl-*sn*-glycerol (IV) for 24 hours. In wild type cells, labeled alkyl [II] and alk-1'-enyl phospholipids [III] were formed. These species with variable side chain length and double bond content were not separated according to the vinyl ether bond but co-eluted together. They were distinguished by the selective lability of [III] to HCl. No pyrenedecanal-dimethylacetal [V] appeared upon acetic acid treatment (upper panel). With HCl, in contrast, alk-1'-enyl ether bonds were cleaved and the pyrenedecanal dimethylacetal [V] appeared (second panel). In *TMEM189*-deficient cells chromatograms of acetic acid treated extracts (third panel) looked similar to those of acetic acid treated wild type cells (upper panel). Upon HCl (bottom panel), however, no pyrenedecanal dimethylacetal [V] was formed, indicating the inability of the cells to form the 1-*O*-alk-1'-enyl ether double bond. *(B)* Quantification of labeled 1-*O*-alk-1'-enyl (full bars) and 1-*O*-alkyl (open bars) phospholipids in HAP1 cells fed with 1-*O*-pyrenedecyl-*sn*-glycerol for 24 hours. Wild type HAP1 cells and *TMEM189*-deficient HAP1 cells were compared to *TMEM189*-deficient HAP1 cells transfected with expression plasmids for the indicated (untagged) proteins preceded by the plus sign. Mean ± SEM for three independent experiments are shown. *(C)* Experiment similar to that shown in *(B)* but with the use of expression plasmids for C-terminally 6xmyc tagged proteins which enabled monitoring of recombinant protein formation in the cells (see Figure S1 above). Mean ± SEM for five independent experiments are shown.

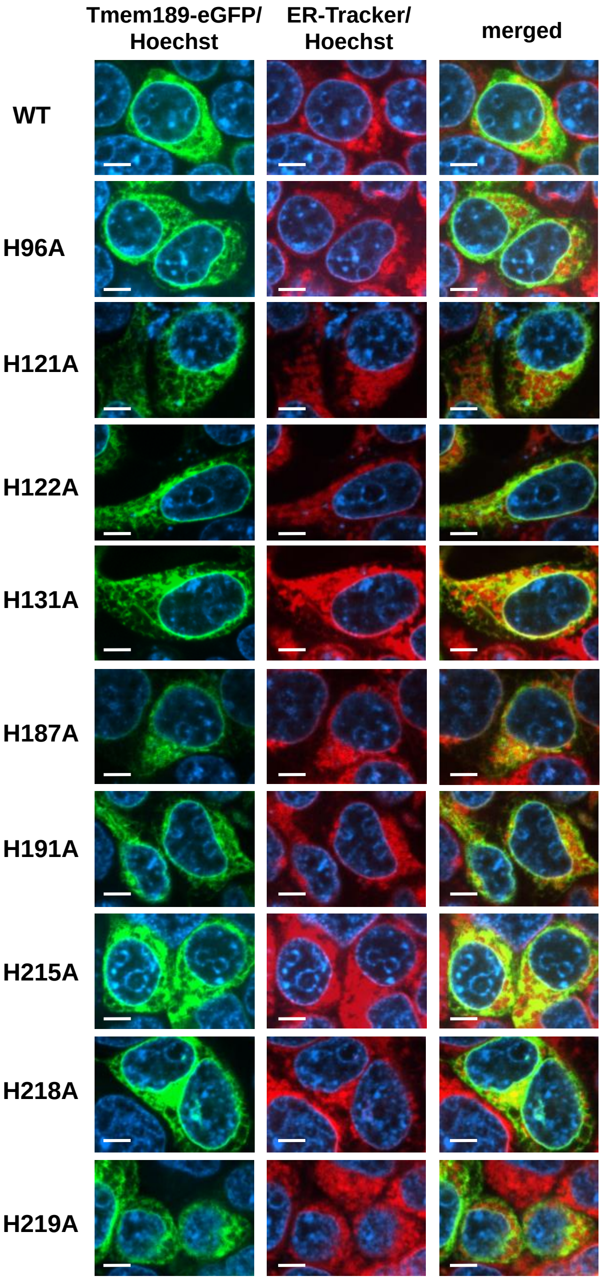

Fig. S3. Localization of expressed TMEM189-GFP fusion proteins in HEK293T cells. HEK293T cells were transfected with plasmids expressing wild type and the indicated mutant TMEM189-GFP fusion proteins and investigated by live confocal microscopy 48 hours post transfection. First column, green fluorescence of the TMEM189-GFP fusion proteins and blue Hoechst 33342 stain of nuclei. Second column, red fluorescence of ER Tracker Red and blue Hoechst 33342 stain of nuclei. Third column, overlay. Scale bar 5 µm. One representative picture of three independent transfection experiments is shown.

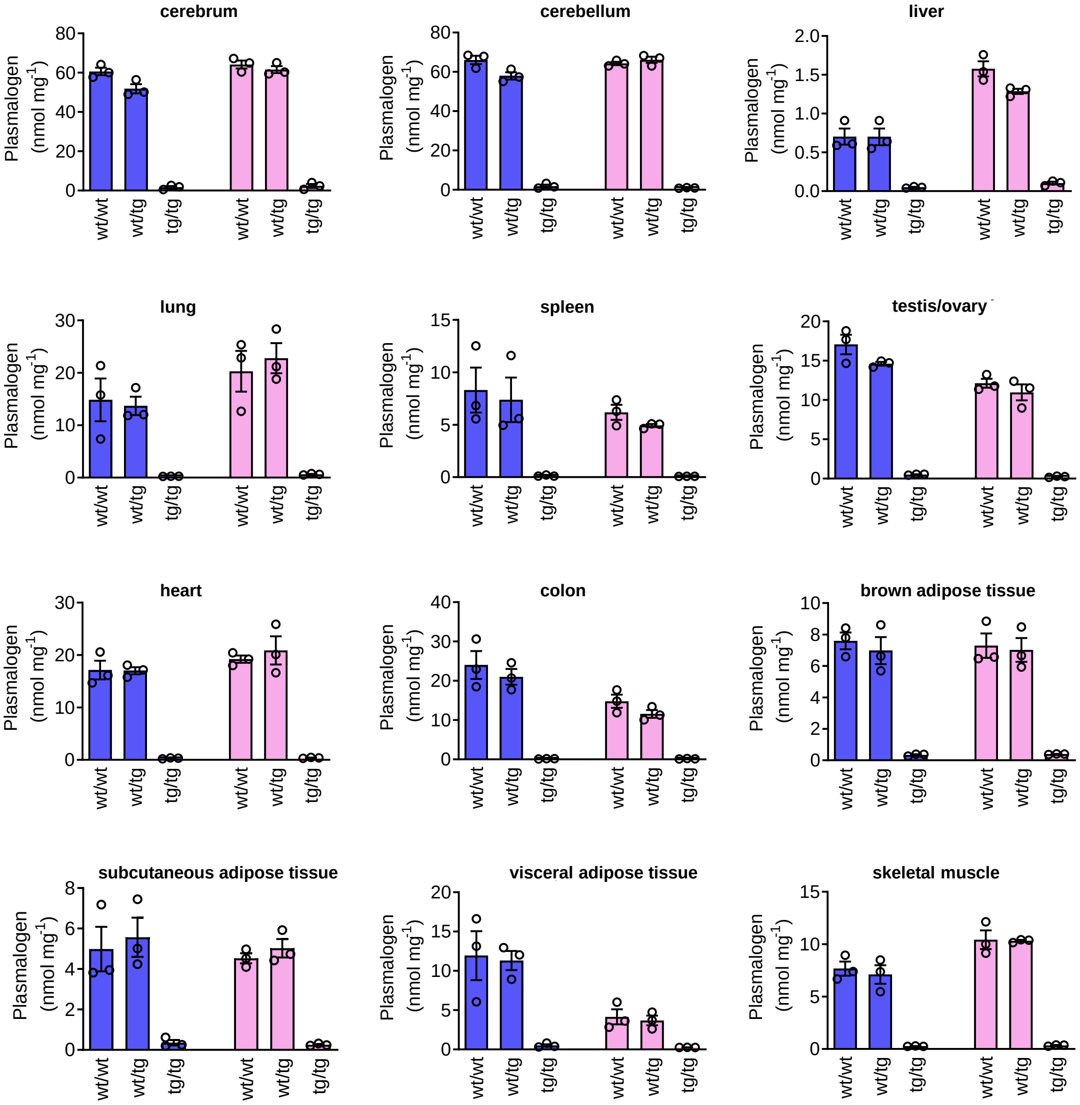

Fig. S4. Plasmalogen content in tissues of mice depending on the *Tmem189* gene locus. Transgene (tg) loci contain an additional cassette downstream exon 2 with a splice acceptor site which causes the formation of a truncated version of the Tmem189 protein fused to a galactosidase protein thereby inactivating the enzymatic activity (8). Lipid extracts of tissues obtained at 8 weeks of age were treated with HCl or acetic acid in presence of dansylhydrazine thus creating fluorescent derivatives from plasmalogens plus free aldehydes (in the presence of HCl), or free aldehydes only (in presence of acetic acid (1)). Free aldehydes were typically less than 1% as compared to plasmalogens and subtracted from the amounts formed in presence of HCl. Results for the analysis of tissues from three male and three female mice (mean ± SEM) are shown. wt, wild type. Blue, male animals, magenta, female animals.

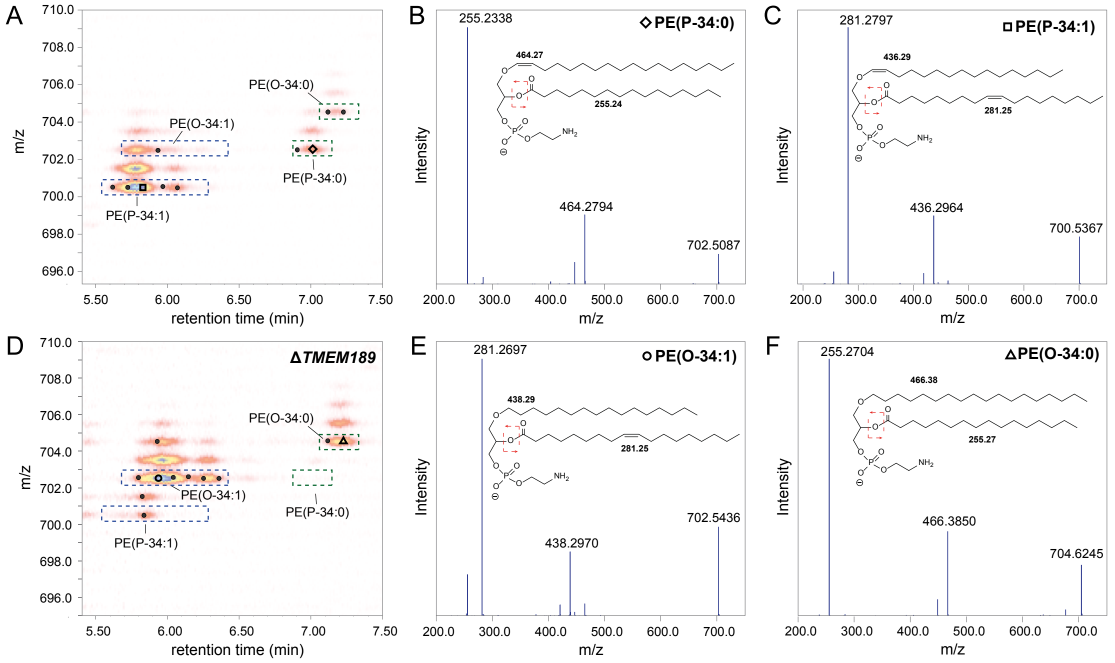

Fig. S5. Quantification of plasmanyl and plasmenyl lipids by means of HPLC-MS/MS. *(A)* 2D illustration of the MS1-data for HAP1 wild type lipid extracts in the retention time range 5.5 - 7.5 min *versus* the mass window 696 – 710 m/z. Color scale corresponds to the respective signal intensities as assigned by Mzmine2. The position of the monoisotopic mass peaks for PE(O-34:1), PE(P-34:1) , PE(O-34:0), and PE(P-34:0) are shown in dashed boxes. In wild type HAP1 cells the plasmenyl species dominated (see Figure 2 of the main manuscript). Fragment spectra were recorded at positions indicated by black dots, and the positions of fragment spectra shown in parts *(B)* and *(C)* of the Figure are indicated by open diamond and open square symbols. *(B)* Fragment spectrum for PE(P-34:0) (diamond). *(C)* Fragment spectrum for PE(P-34:1) (square). *(D)* 2D illustration of the MS1-data for lipid extracts of *TMEM189*-deficient HAP1 cells in the retention time range 5.5 - 7.5 min *versus* the mass window 696 – 710 m/z. Color scale corresponds to the respective signal intensities as assigned by Mzmine2. As also measured by an independent method (fluorescent aldehyde derivatives quantified by HPLC and fluorescence detection (Figure 1H of the main manuscript)), plasmenyl species were strongly reduced in *TMEM189*-deficient HAP1 cells. Respective plasmanyl species accumulated instead, which is reflected in the peak pattern shown here. The position of the monoisotopic mass peak for PE(O-34:1), PE(P-34:1) , PE(O-34:0), and PE(P-34:0) are shown in dashed boxes. Position of fragment spectra shown in parts *(E)* and *(F)* of the Figure are indicated by open circle and triangle symbols. *(E)* Fragment spectrum for PE(O-34:1) (circle) and *(F)* fragment spectrum for PE(O-34:1) (triangle).

SI References

1. E.R. Werner, M.A. Keller, S. Sailer, D. Seppi, G. Golderer, *et al.* (2018) A novel assay for the introduction of the vinyl ether double bond into plasmalogens using pyrene-labeled substrates. *J Lipid Res* 59(5):901-909.
2. K. Tamura, G. Stecher, D. Peterson, A. Filipski, S. Kumar (2013) MEGA6: Molecular Evolutionary Genetics Analysis version 6.0. *Mol Biol Evol* 30(12):2725-2729.
3. V.A. Blomen, P. Majek, L.T. Jae, J.W. Bigenzahn, J. Nieuwenhuis, *et al.* (2015) Gene essentiality and synthetic lethality in haploid human cells. *Science* 350(6264):1092-1096.
4. M.A. Keller, K. Watschinger, K. Lange, G. Golderer, G. Werner-Felmayer, *et al.* (2012) Studying fatty aldehyde metabolism in living cells with pyrene-labeled compounds. *J Lipid Res* 53(7):1410-1416.
5. G. Oemer, K. Lackner, K. Muigg, G. Krumschnabel, K. Watschinger, *et al.* (2018) Molecular structural diversity of mitochondrial cardiolipins. *Proc Natl Acad Sci U S A* 115(16):4158-4163.
6. J. Folch, M. Lees, G.H. Sloane Stanley (1957) A simple method for the isolation and purification of total lipides from animal tissues. *J Biol Chem* 226(1):497-509.
7. T. Pluskal, S. Castillo, A. Villar-Briones, M. Oresic (2010) MZmine 2: modular framework for processing, visualizing, and analyzing mass spectrometry-based molecular profile data. *BMC Bioinformatics* 11:395.
8. J.K. White, A.K. Gerdin, N.A. Karp, E. Ryder, M. Buljan, *et al.* (2013) Genome-wide generation and systematic phenotyping of knockout mice reveals new roles for many genes. *Cell* 154(2):452-464.
